# Interaction of the Trans-Frame Potyvirus Protein P3N-PIPO with Host Protein PCaP1 Facilitates Potyvirus Movement

**DOI:** 10.1371/journal.ppat.1002639

**Published:** 2012-04-12

**Authors:** Paramasivan Vijayapalani, Masayoshi Maeshima, Nahoko Nagasaki-Takekuchi, W. Allen Miller

**Affiliations:** 1 Plant Pathology and Microbiology Department, Center for Plant Responses to Environmental Stresses, Iowa State University, Ames, Iowa, United States of America; 2 Laboratory of Cell Dynamics, Graduate School of Bioagricultural Sciences, Nagoya University, Nagoya, Japan; University of Kentucky, United States of America

## Abstract

A small open reading frame (ORF), *pipo*, overlaps with the P3 coding region of the potyviral polyprotein ORF. Previous evidence suggested a requirement for *pipo* for efficient viral cell-to-cell movement. Here, we provide immunoblotting evidence that the protein PIPO is expressed as a trans-frame protein consisting of the amino-terminal half of P3 fused to PIPO (P3N-PIPO). P3N-PIPO of *Turnip mosaic virus* (TuMV) fused to GFP facilitates its own cell-to-cell movement. Using a yeast two-hybrid screen, co-immunoprecipitation assays, and bimolecular fluorescence complementation (BiFC) assays, we found that P3N-PIPO interacts with host protein PCaP1, a cation-binding protein that attaches to the plasma membrane via myristoylation. BiFC revealed that it is the PIPO domain of P3N-PIPO that binds PCaP1 and that myristoylation of PCaP1 is unnecessary for interaction with P3N-PIPO. In *PCaP1* knockout mutants (*pcap1*) of *Arabidopsis*, accumulation of TuMV harboring a GFP gene (TuMV-GFP) was drastically reduced relative to the virus level in wild-type plants, only small localized spots of GFP were visible, and the plants showed few symptoms. In contrast, TuMV-GFP infection in wild-type *Arabidopsis* yielded large green fluorescent patches, and caused severe stunting. However, viral RNA accumulated to high level in protoplasts from *pcap1* plants indicating that PCaP1 is not required for TuMV RNA synthesis. In contrast to TuMV, the tobamovirus *Oilseed rape mosaic virus* did not require PCaP1 to infect *Arabidopsis* plants. We conclude that potyviral P3N-PIPO interacts specifically with the host plasma membrane protein PCaP1 to participate in cell-to-cell movement. We speculate that PCaP1 links a complex of viral proteins and genomic RNA to the plasma membrane by binding P3N-PIPO, enabling localization to the plasmodesmata and cell-to-cell movement. The *PCaP1* knockout may contribute to a new strategy for recessive resistance to potyviruses.

## Author Summary

The Potyviridae is the largest and most economically important family of plant viruses. A key step in the life cycle of all plant viruses is transport of the viral genome through the plasmodesmata, highly regulated channels that connect cells. While the mechanisms of cell-to-cell movement of many plant viruses have been characterized, our understanding of Potyviridae movement is lacking. The viral RNA genome is transported to the plasmodesmata by a complex of viral proteins including a recently discovered protein, P3N-PIPO which is encoded in two reading frames. The details of this localization process are unclear. Here, we identify a potential missing link that suggests how the potyviral movement complex may anchor to the plasma membrane including in the plasmodesmata. The host protein PCaP1, a divalent cation-binding plasma membrane protein, binds the P3N-PIPO protein of *Turnip mosaic virus* (TuMV). Both proteins were detected in the plasma membrane and plasmodesmata. *Arabidopsis* plants lacking PCaP1 allowed TuMV RNA replication but showed inefficient TuMV movement, reduced TuMV accumulation, and had greatly attenuated symptoms. However, these plants allowed normal infection by a tobamovirus. Thus, mutation of the *PCaP1* gene may contribute to breeding potyvirus-resistant crops.

## Introduction

To spread beyond the initially infected cell, the genome of a plant virus must move through the plasmodesmata, which are narrow tunnels through the impervious cell wall that connect cytoplasm, endoplasmic reticulum and plasma membrane between adjoining cells [1], [2]. Viral nucleic acid is too large to move through the plasmodesmata on its own, so viruses have evolved movement proteins (MPs) that interact with host proteins to modify the plasmodesmata and transport the viral genome from cell-to-cell [3], [4], [5], [6], [7]. Viruses have evolved diverse types of MPs such as the 30K-type MP of *Tobacco mosaic virus* (TMV) and related viruses [4], the triple gene block proteins of Potex-, Hordei- and other viruses [5], and the tubule-forming MPs of the Secoviridae, Bromoviridae and Caulimoviridae [8]. However the cell-to-cell movement mechanism of the largest family of plant viruses, the Potyviridae, falls into no previously known category, and is poorly understood. No dedicated MP has been identified but many viral proteins with other known functions have been reported to participate in potyvirus movement. Here, we describe the interaction of a novel potyviral protein, called P3N-PIPO, with a previously unrecognized host protein that provides a key insight into the cell-to-cell movement process of the potyviruses.

**Figure 1:**
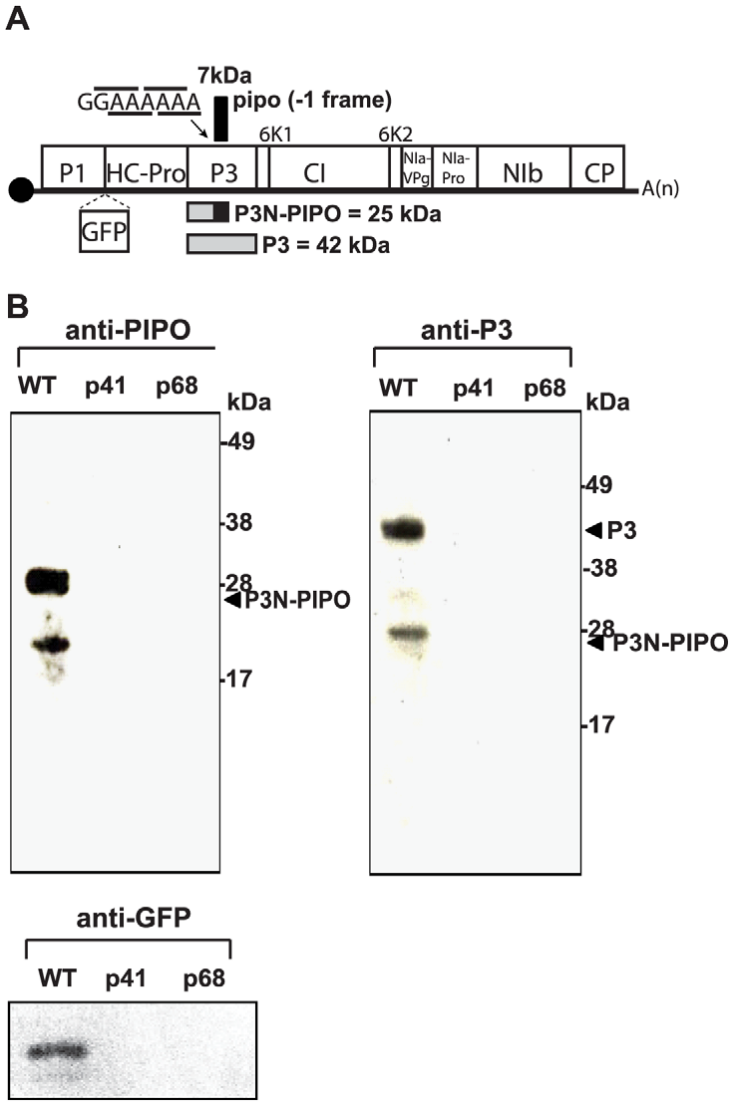
Genome map of TuMV-GFP and expression of P3N-PIPO *in planta*. (A) Genome map of TuMV-GFP. Mature proteins processed from the proteolytic cleavage of the large polyprotein are indicated in boxes. *pipo* ORF is indicated above the polyprotein ORF with putative frameshift sequence indicated and bars showing codons in the polyprotein ORF (below) and *pipo* ORF (above). Sizes of P3N-PIPO and P3 proteins are indicated below the regions that code for them. P3-derived portion is shaded gray; PIPO-derived portion is in black. Position of inserted GFP coding insertion is indicated by dashed lines. Solid circle indicates VPg at 5′ end; (A)n indicates poly(A) tail. (B) Immunodetection of P3N-PIPO and P3 in TuMV-GFP-infected *Arabidopsis*. Total protein was extracted from the infected leaves at 14 days post inoculation (dpi), separated in either 4–12% NuPAGE Bis-Tris gel (Life Technologies) (for P3-N-PIPO and P3 detections) or in 4–12% Novex Tris-glycine gel (for GFP detection), blotted onto PVDF membrane and probed with anti-PIPO, anti-P3 or anti-GFP antibody and detected by ECL-plus Western reagents. Positions of protein mobility markers in kilodaltons (kDa) are indicated at right. Lanes indicate total protein from plants inoculated with wild-type (WT) TuMV-GFP, or *pipo* knockout mutants of TuMV-GFP (p41 and p68). These mutants differ from WT TuMV-GFP by single point mutations that introduce stop codons into the *pipo* ORF: CGA→UGA and GGA→UGA at bases 3103 and 3130 in mutants p41 and p68, respectively (*pipo*-frame codons shown). These mutations do not alter the amino acid sequence of the overlapping P3 region of the polyprotein. Note that putative P3N-PIPO migrates more slowly (∼28 kDa) than its predicted molecular weight (∼25 kDa).

Potyviruses have a positive-strand, ∼10 kb RNA genome that encodes a large polyprotein precursor which is processed into about ten multifunctional proteins (Fig. 1A) by a series of viral proteases [9], [10]. Recently, a small open reading frame (ORF) termed *pipo*, predicted to encode a ∼7 kDa protein was discovered to overlap with the P3 coding region in all members of the Potyviridae family [11]. Mutations that knockout *pipo* expression in *Turnip mosaic virus* (TuMV) rendered the virus noninfectious in *Nicotiana benthamiana* [11]. Immunodetection of the *pipo*-encoded protein in TuMV infected cells revealed a ∼25 kDa polypeptide, consistent with expression of *pipo* as a translational fusion with the N-terminus of P3 [11]. We call this protein P3N-PIPO. P3N-PIPO is probably translated by ribosomal frameshifting from the P3 coding region into the *pipo* ORF at a highly conserved G_1–2_A_6–7_ motif at the beginning of the *pipo* ORF [11].

Proteins implicated in potyviral cell-to-cell movement include helper component-protease (HC-Pro) [12], coat protein (CP) [13], [14], [15], [16], [17], genome-linked protein (VPg) [18], and cylindrical inclusion protein (CI) [17], [19], [20]. Indirect evidence suggests P3N-PIPO is also required for efficient cell-to-cell movement of the virus but not for RNA replication. Choi et al. [21] reported that synonymous mutations in the P3 cistron, but which altered the *pipo* ORF (which wasn't known at the time) of *Wheat streak mosaic virus* (WSMV) disrupted the movement of WSMV in plants. In the *Soybean mosaic virus* (SMV) genome, premature stop codons within *pipo* or mutations in the conserved G_1–2_A_6–7_ motif that did not alter the P3 amino acid sequence, restricted SMV accumulation to small infection foci in the inoculated leaves [22]. P3N-PIPO has been shown to interact with the TuMV CI protein and directs it to the plasmodesmata via the secretory pathway [23]. The CI protein binds the virion [24] and colocalizes with the CP on conical structures at the plasmodesmata [17], and binds with the CP bound to the viral genomic RNA, probably in the form of intact virion [13], [14].

The nature of host factors involved in the intercellular trafficking of potyviruses is poorly understood. A number of host proteins such as calmodulin and calmodulin-related protein [25], RING finger protein HIP1 [26], 20S proteasome and its four subunits [27], [28], chloroplast division-related factor NtMinD [29], chloroplast precursor of ferredoxin-5 [30] and calreticulin [12], have been reported to interact with HC-Pro of various potyviruses. However, these proteins may be important for functions of HC-Pro other than movement, such as suppression of host antiviral silencing, aphid transmission, or cleavage of the polyprotein. The CP of *Potato virus Y* (PVY) interacts with a subset of tobacco DnaJ-like proteins, NtCPIPs that act as important susceptibility factors during PVY infection that may be involved in virion assembly and/or movement [16]. Eukaryotic translation initiation factor eIF4E [31] and a cysteine-rich protein [18] have been identified as susceptibility factors supporting potyvirus movement.

To better understand the function of P3N-PIPO in potyvirus infection including cell-to-cell movement, we identified a host protein with which it interacts. We used a yeast two-hybrid screen to identify a hydrophilic plasma membrane-associated cation binding protein, PCaP1 that interacts with the P3N-PIPO of TuMV. The specificity of P3N-PIPO and PCaP1 interaction was validated *in planta* where P3N-PIPO was found to colocalize with PCaP1 in the plasma membrane and plasmodesmata. Virus accumulation, movement and disease symptoms were dramatically reduced in an *Arabidopsis PCaP1* knockout. Together, these results suggest that PCaP1 represents a new type of plant protein required for efficient infection by potyviruses, and which may participate in intercellular trafficking of potyviruses.

## Results

### PIPO is expressed as a fusion with the N-terminus of P3 in TuMV infected plants

Previously, Chung et al. [11] showed that antibody against epitopes on PIPO detected only a ∼25 kDa protein in TuMV infected *N. benthamiana* plants. No 7 kDa protein, the predicted size of free PIPO, was detected. The simplest explanation was that PIPO is expressed as a translational fusion with the N-terminus of P3 which would be a ∼25 kDa protein, we call P3N-PIPO. We predict that P3N-PIPO is translated by ribosomal shifting from the P3 coding region into the *pipo* ORF at the highly conserved G_1–2_A_6–7_ motif at the beginning of the *pipo* ORF [11]. To determine if this putative P3N-PIPO protein is expressed as predicted, total protein from *Arabidopsis* leaves infected with GFP-tagged TuMV (TuMV-GFP) (Fig. 1A) was immunoblotted using antibody targeting an N-terminal region of P3 or antibody targeting a PIPO epitope. PIPO specific antibody recognized two polypeptides, a polypeptide that migrated as ∼28 kDa and a smaller unexpected polypeptide of ∼18 kDa (Fig. 1B). The larger protein is P3N-PIPO, as the protein migrates somewhat slower than its predicted size (∼25 kDa) as detected previously in infected *N. benthamiana* [11]. The antibody against the N-terminus of P3 recognized proteins of ∼42 kDa and ∼28 kDa that correspond to the predicted molecular masses of P3 and P3N-PIPO, respectively (Fig. 1B). Antibodies targeting PIPO or the N-terminus of P3 did not recognize any protein from uninoculated plants or plants inoculated with the *pipo* knockout mutants of TuMV-GFP (p41, p68; [11]) (Fig. 1B). Immunodetection of GFP in plants infected with wild-type (WT) TuMV-GFP confirmed the viral infection (Fig. 1B). Recognition of a polypeptide that migrates at ∼28 kDa by anti-PIPO and anti-P3 antibodies, and absence of a 7 kDa protein expected for free PIPO, indicates that the protein indeed consists of a fusion of the N-terminus of P3 with PIPO, i.e., P3N-PIPO.

### P3N-PIPO-GFP facilitates its own cell-to-cell movement

**Figure 2:**
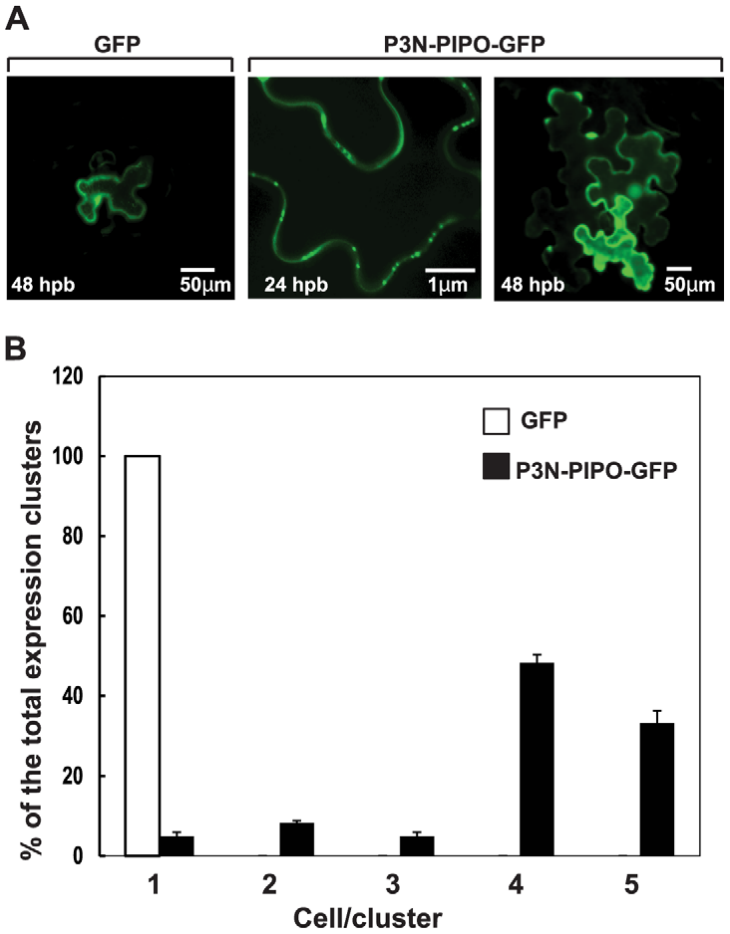
Cell-to-cell movement of P3N-PIPO-GFP in *N. benthamiana* leaves. (A) Images of epidermal cells expressing GFP alone or fused to P3N-PIPO (P3N-PIPO-GFP) at indicated hours post biolistic bombardment (hpb). Images are projections of single confocal section. (B) Quantification of cell-to-cell movement of GFP and P3N-PIPO-GFP. At least 160 GFP-expressing clusters in each bombarded sample were analyzed at 24 hpb and 48 hpb. At 48 hpb the number of cells in each cluster was counted, followed by statistical evaluation by the unpaired Student's *t*-test (P = 0.151). Error bars represent standard deviations.

Many MPs can move from cell-to-cell in the absence of their viral RNA cargo [7], [32]. Therefore, we tested potential of P3N-PIPO for cell-to-cell movement. We constructed a gene that expresses a PIPO-GFP fusion in-frame with the N terminus of P3 to generate P3N-PIPO-GFP. This construct contains one nucleotide insertion in the putative frameshift site (GGAAAAAA) to allow expression of P3N-PIPO without the need for frameshifting. However, we do not know the exact frameshift site, so the artificial in-frame construct may differ by one or two amino acids from the natural P3N-PIPO translation product. Detached *N. benthamiana* leaves were bombarded with plasmids encoding GFP or P3N-PIPO-GFP, each driven by a 35S promoter and terminating with a *nos* signal. By 48 hours post bombardment (hpb) with plasmid encoding GFP alone, green fluorescence was confined to single cells and never diffused to adjacent cells (Fig. 2). P3N-PIPO-GFP accumulated at the periphery of the cell as punctate inclusions at 24 hpb, and by 48 hpb, not only did the bombarded cells fluoresce, but many of the adjacent cells also fluoresced green (Fig. 2A). Out of 160 fluorescent cell clusters observed for P3N-PIPO-GFP movement, 130 clusters had 4–5 cells fluorescent cells, indicating significant cell-to-cell movement (Fig. 2B). Thus, the P3N-PIPO portion of P3N-PIPO-GFP allowed the protein to move to adjacent cells, as has been shown for other viral MPs [32], [33].

### P3N-PIPO interacts with host protein PCaP1 in yeast cells

To identify the cellular proteins that interact with P3N-PIPO, a cDNA library from *A. thaliana* was screened, utilizing the GAL4-based yeast two-hybrid (Y2H) system with TuMV P3N-PIPO as bait. As a result of sequential screening steps, ten positive clones were isolated and sequenced. Out of the ten clones, five encoded a protein known as PCaP1 (TAIR accession AT4G20260; GenBank accession NM_118145). Since PCaP1 showed the strongest interaction with P3N-PIPO in the yeast cells compared to other interactors (data not shown), PCaP1 was chosen for further investigation.

**Figure 3:**
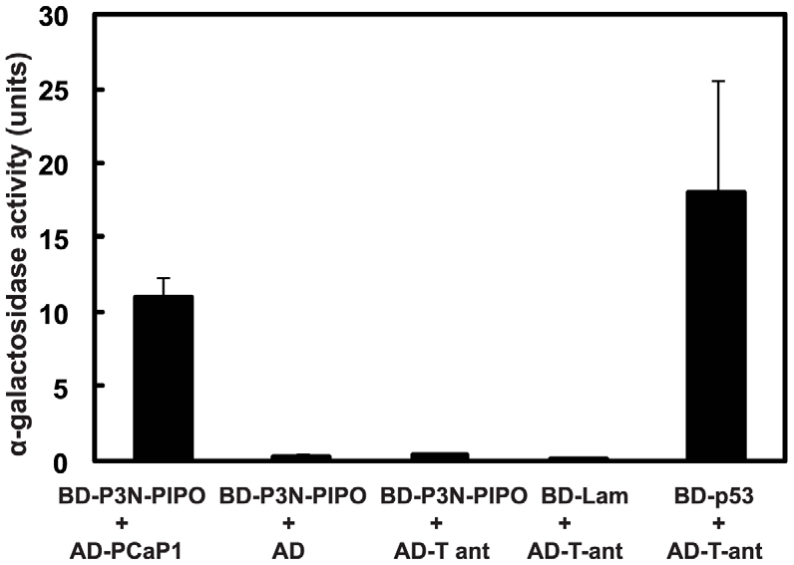
Interaction of P3N-PIPO with Arabidopsis PCaP1 in yeast cells. Yeast strain Y2HGold co-transformed with the BD-P3N-PIPO bait vector in combination with either the PCaP1 prey vector (AD-PCaP1), empty prey vector (AD), or T-antigen prey vector (AD-T antigen), and plated on non-selective medium (SD/−Leu/−Trp/+X-α-Gal). Individual colonies of each combination were picked to quantify interaction by α-galactosidase activity in liquid medium. Data are average of eight replicates from three independent experiments. Error bars represent standard deviations. Negative control: BD-lam and AD-T-ant, positive control: BD-murine p53 and AD-T-ant, lam (human lamin C), SV-40 large T-antigen (T-ant), binding domain (BD), activation domain (AD).

Interaction between P3N-PIPO and PCaP1 was verified in yeast transformants expressing P3N-PIPO bait in combination with either the rescued prey plasmid encoding PCaP1, empty prey vector, or prey plasmid encoding unrelated protein SV40 large T-antigen (Clontech). Only colonies co-transformed with P3N-PIPO and PCaP1 expression plasmids expressed α- galactosidase and appeared blue on low stringency medium SD/−Leu/−Trp/+X-α-Gal and could grow on high stringency selective medium SD/−Leu/−Trp/−Ade/−His, confirming the protein-protein interaction. Interaction between P3N-PIPO and PCaP1 induced high expression of the α-galactosidase gene in liquid medium, as did the interaction of p53 with large T-antigen which was used as a positive control (Fig. 3). P3N-PIPO did not interact to activate α-galactosidase synthesis in cells harboring empty vector or expressing T-antigen.

PCaP1 is a hydrophilic cation binding protein (*M_r_* 24.5 kDa, pI 4.6) associated with the plasma membrane [34]. It lacks a transmembrane domain and anchors to the plasma membrane via myristoylation of a glycine residue. The *PCaP1* gene is present as a single copy in *Arabidopsis*. Amino acid sequence alignment shows that *Arabidopsis* PCaP1 shares up to 67% sequence identity with ortholog in dicots including potyvirus hosts and up to 54% with ortholog in monocot species (Fig. S1). An ortholog was also detected in a gymnosperm but not in lower plants.

### PCaP1 interacts with P3N-PIPO *in planta*

**Figure 4:**
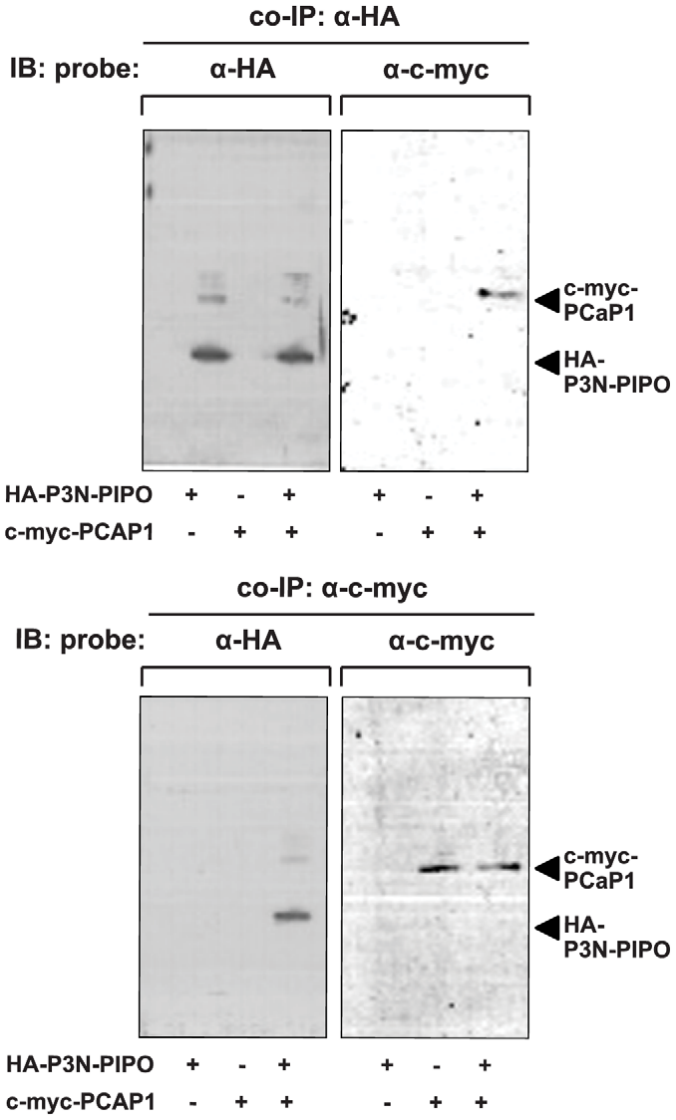
Co-immunoprecipitation of P3N-PIPO and PCaP1 expressed *in planta*. Proteins from crude extracts of *N. benthamiana* leaves (2 days post agroinfiltration) that co-expressed HA-P3N-PIPO and c-myc-PCaP1, or expressed HA-P3N-PIPO or c-myc-PCaP1 only were pulled-down using anti-HA (top panels) or anti-c-myc (bottom panels) antibodies, separated by 4–12% Novex Tris-Glycine PAGE, electroblotted onto PVDF membrane and probed with anti-HA or anti-c-myc antibody as indicated. Recognition of HA-P3N-PIPO and c-myc-PCAP1 are shown at right. Immunoblotting (IB).

To determine whether P3N-PIPO and PCaP1 interact *in planta*, HA-tagged P3N-PIPO and c-myc-tagged PCaP1 were co-expressed transiently in *N. benthamiana* leaves and total protein was extracted and subjected to co-immunoprecipitation (co-IP) with either anti-HA or anti-c-myc antibodies. Proteins bound to the matrix were eluted and immunodetected with either anti-HA or anti-c-myc antibodies (Fig. 4). Anti-c-myc antibodies detected c-myc-PCaP1 among proteins pulled-down with anti-HA antibodies (Fig. 4, top right panel), and anti-HA antibodies detected HA-P3N-PIPO among proteins pulled-down by anti-c-myc antibodies (Fig. 4, bottom left panel), in both cases only when plants were co-infiltrated with agrobacterium harboring both the plasmids. Thus, binding of HA-P3N-PIPO to c-myc-PCaP1 is evident from the immunodetection of both proteins that were captured as a complex with anti-HA antibody or anti-c-myc antibody. Expression and immunoprecipitation of HA-P3N-PIPO and c-myc-PCaP1 were confirmed by immunoblotting (Fig. 4, panels at top left and bottom right).

**Figure 5:**
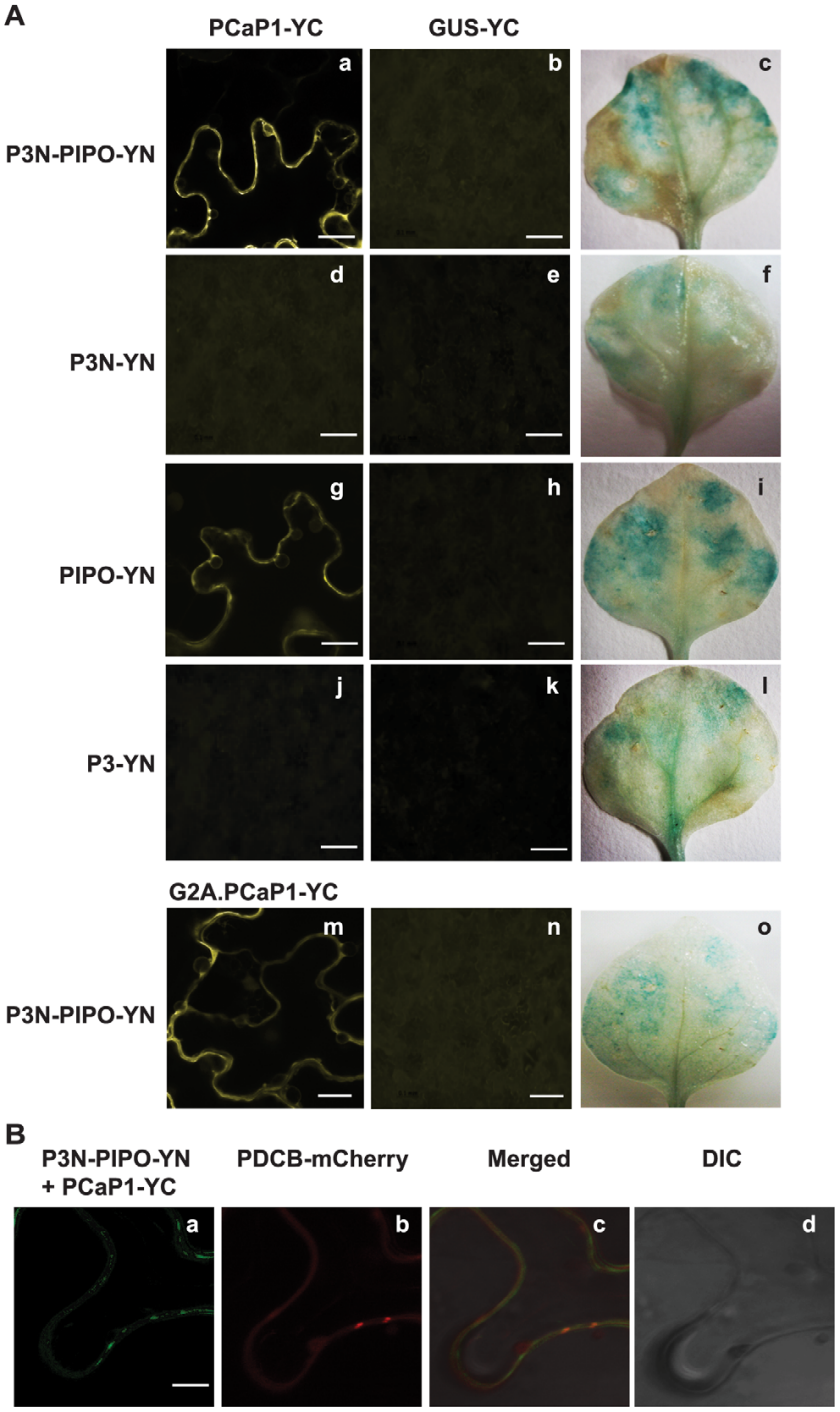
Bimolecular fluorescence complementation assay of P3N-PIPO, P3N, PIPO and P3 with wild-type and mutant PCaP1 in *N. benthamiana*. (A) Interactions of P3N-PIPO-YN, P3N-YN, PIPO-YN or P3-YN with PCaP1-YC, or P3N-PIPO-YN with PCaP1 containing a G to A amino acid substation at position two (G2A.PCaP1-YC) were analyzed at about 38 h post agroinfiltration (hpa) (panels a, d, g, j and m). P3N-PIPO-YN, P3N-YN, PIPO-YN or P3-YN interaction with GUS-YC are in panels b, e, h, k and n. Expression of GUS-YC was confirmed in all pair-wise interactions by histochemical staining (panels c, f, i, l and o). YN, N-terminal half of citrine; YC, C-terminal half of citrine; scale bar = 25 µm. (B) Co-localization of P3N-PIPO-YN, PCaP1-YC and PDCB1-mCherry in plasmodesmata (38 hpa). Interaction of P3N-PIPO-YN and PCaP1-YC (color changed digitally to green fluorescence, panel a). Localization of PDCB1-mCherry (panel b). Merged citrine signals and mCherry signals show colocalization of P3N-PIPO, PCaP1 and PDCB1-mCherry as orange spots (panel c). Nomarski DIC image of the same cell in panels a-c (panel d). All images are single confocal sections. Scale bar ∼10 µm.

To determine whether the interaction between P3N-PIPO and PCaP1 *in planta* is direct or mediated by other cellular protein(s), bimolecular fluorescence complementation (BiFC) assays were conducted in *N. benthamiana* leaves (Fig. 5). To this end, chimeric constructs expressing P3N-PIPO fused to the N-terminal half of the yellow fluorescent protein citrine (P3N-PIPO-YN) and PCaP1 fused to the C-terminal half of citrine (PCaP1-YC) were transiently co-expressed via agroinfiltration, and fluorescence was detected by confocal microscopy. Yellow fluorescence was detected in cells co-expressing P3N-PIPO-YN and PCaP1-YC (Fig. 5A, panel a), indicating direct interaction of P3N-PIPO and PCaP1. Expression of P3N-PIPO-YN alone, PCaP1-YC alone, or P3N-PIPO-YN with GUS-YC served as negative controls. No fluorescence was detected in cells agroinfiltrated with any of these constructs (Fig. 5A, panel b, and data not shown).

We next determined whether the P3N domain (N terminus of P3) or the PIPO domain of P3N-PIPO interacts with PCaP1 by testing the interaction of P3N-YN or PIPO-YN with PCaP1-YC (Fig. 5A, panels d and g). Additionally, we tested for interaction of full-length P3 fused to YN (P3-YN) with PCaP1-YC (Fig. 5A, panel j). As negative controls, each of the fusion proteins was expressed alone or in pairwise combination with GUS-YC (Fig. 5A, panels e, h and k). Citrine fluorescence was observed in cells co-expressing PIPO-YN and PCaP1-YC (Fig. 5A, panel g), but cells expressing P3N-YN and PCaP1-YC, or P3-YN and PCaP1-YC yielded no fluorescent signal (Fig. 5A, panels d and j). We conclude that the PIPO domain is the part of P3N-PIPO that interacts with PCaP1, and that PIPO alone is sufficient to interact with PCaP1.

PCaP1 was shown previously to be myristoylated at the glycine residue at position 2 [34]. This anchors PCaP1 in the plasma membrane. To determine if the interaction between P3N-PIPO and PCaP1 requires myristoylation of PCaP1, we tested interaction of P3N-PIPO-YN with YC-fused mutant PCaP1 lacking the myristoylation site by a glycine to alanine substitution at position 2 (G2A.PCaP1-YC). Co-expression of P3N-PIPO-YN and G2A.PCaP1-YC revealed yellow fluorescence (Fig. 5A, panel m) indicating that myristoylation, and thus membrane binding, of PCaP1 is apparently not necessary for binding by P3N-PIPO. Expression of P3N-PIPO-YN or G2A.PCaP1-YC alone (data not shown), or P3N-PIPO-YN coexpressed with GUS-YC (Fig. 5, panel n) gave no fluorescent signal. GUS-YC expression in the negative controls was confirmed by histochemical staining (Fig. 5A, panels c, f, i, l and o). In summary, the interaction between the P3N-PIPO and PCaP1, identified first in the Y2H assay, was confirmed by co-IP and BiFC, which also demonstrated a direct physical interaction between the PIPO domain of P3N-PIPO and PCaP1 *in planta*.

Previous work provided evidence of co-localization of P3N-PIPO and viral CI protein in plasmodesmata [23]. Thus, we asked whether P3N-PIPO and PCaP1 colocalize in the plasmodesmata. To this end, we used the plasmodesmata callose binding protein PDCB1 fused to mCherry (provided by Dr. Andy Maule; John Innes Center, UK) [35] as a plasmodesmata marker in the BiFC assay (Fig. 5B, panels a–d). The orange punctate structures in the merged images (Fig. 5B, panel c) indicate interacting P3N-PIPO-YN and PCaP1-YC co-localizing with the PDCB1-mCherry, revealing the presence of P3N-PIPO and PCaP1-YC in the plasmodesmata. Note that the yellow color of citrine was digitally altered to green to optimize merged images. However, it is clear that P3N-PIPO-PCaP1 complexes are also present outside of the plasmodesmata. (Compare Fig. 5B panels a and b.)

### Knockout of *PCaP1* inhibits TuMV cell-to-cell movement and accumulation in plants

**Figure 6:**
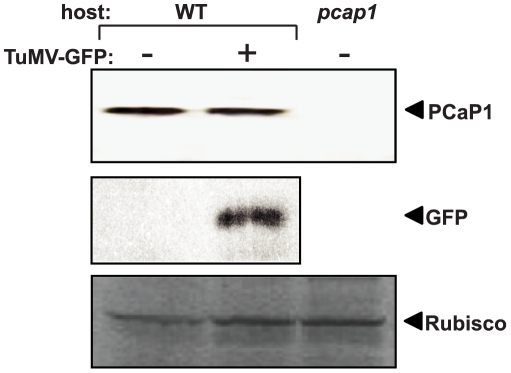
Immunodetection of PCaP1 in wild-type and *PCaP1* knockout *Arabidopsis* plants. Total soluble proteins from leaves collected at 14 dpi were separated by 4–12% Novex Tris-glycine PAGE, blotted onto PVDF membrane, probed with anti-PCaP1 antibody or anti-GFP antibody and detected by ECL-Plus Western reagents. Samples were from mock inoculated or TuMV-GFP infected wild-type (WT) or *PCaP1* knockout (*pcap1*) plants. Equal loading of proteins was verified by similar levels of Coomassie staining of Rubisco protein (bottom panel).

As PCaP1 interacts specifically with the virally encoded P3N-PIPO, it is possible that the level of PCaP1 changes in response to TuMV infection. This was analyzed by immunodetection of PCaP1 in TuMV-GFP-infected vs healthy *Arabidopsis* plants (Fig. 6). In WT plants, anti-PCaP1 antibody recognized endogenous PCaP1 as a single polypeptide, confirming the specificity of the antibody. PCaP1 was detected at similar levels in both healthy and infected plants. TuMV-GFP infection was confirmed by immunodetection of expressed GFP. To test whether PCaP1 plays a key role in potyvirus infection, homozygous *PCaP1* knockout *Arabidopsis* line (SALK_022955; *pcap1*) was used. Insertion of the T-DNA at an intron as a single copy was verified. *pcap1* plants did not differ phenotypically from the WT plants and had normal fecundity. No PCaP1 protein was detectable by immunoblot in *pcap1* plants (Fig. 6), confirming that *pcap1* plants express no PCaP1.

**Figure 7:**
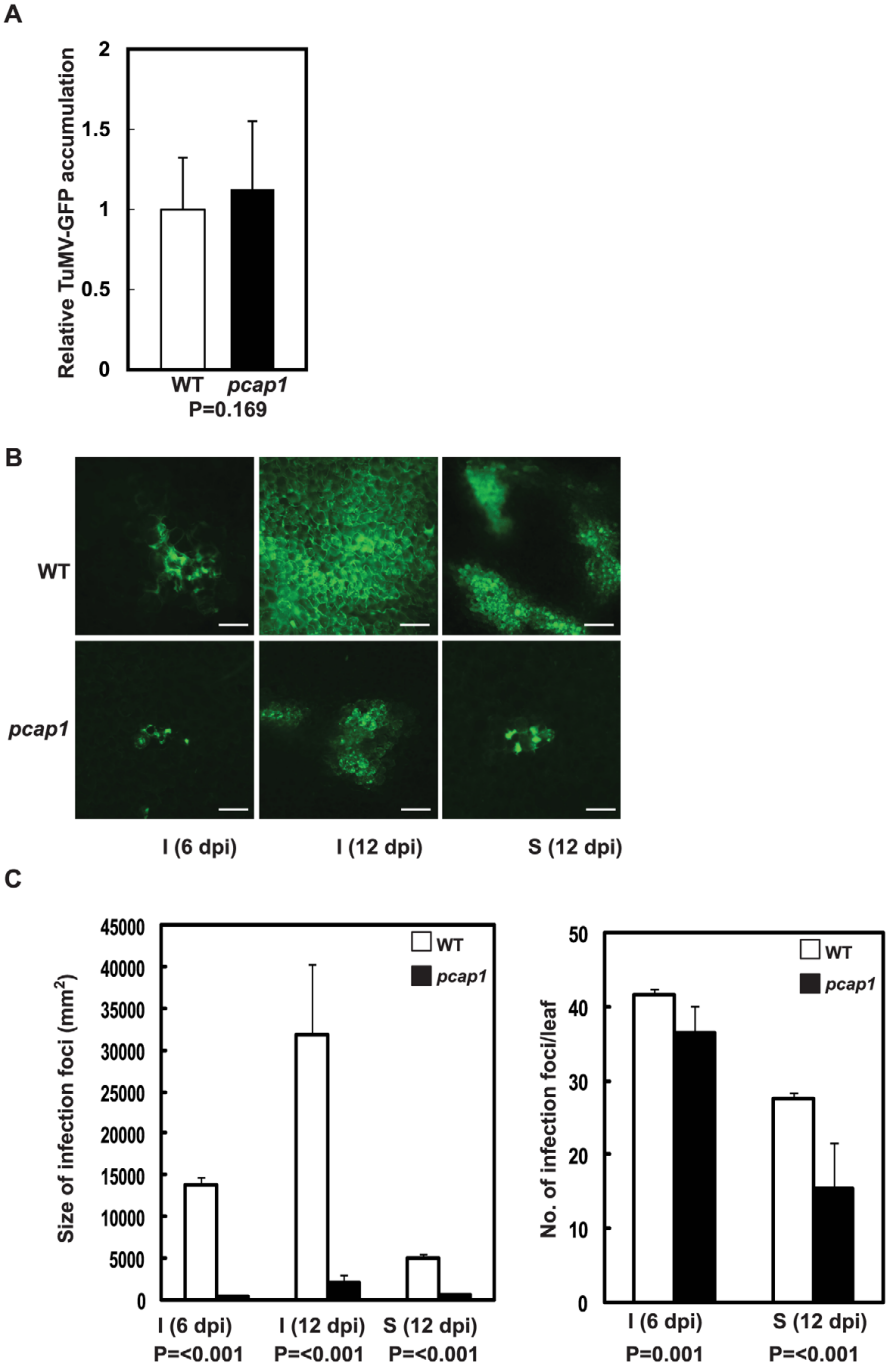
TuMV-GFP infection in wild type and *pcap1 Arabidopsis* plants. (A) qRT-PCR quantification of TuMV-GFP RNA in inoculated leaves of indicated plant lines at 3 dpi. The values were normalized to the amounts of *Actin8* transcript in the same sample. Data are averages of three independent experiments, each consisting of five technical replicates and statistical significance was analyzed by the unpaired Student's *t*-test (P = 0.169). Error bars represent standard deviations. (B) TuMV-GFP infection foci in leaves analyzed by epifluorescence microscopy. Bars = 50 µm. (C) Quantification of size and number of TuMV-GFP infection foci. The data are averages of four independent experiments and each consisting of at least eight replicates. Error bars represent standard deviations. Statistical significance of difference in the size and number of the infection foci between WT and *pcap1* was analyzed by the unpaired Student's *t*-test and the calculated P values are indicated. Wild-type (WT), PCaP1 knocout (*pcap1*), inoculated leaf (I), uninoculated second cauline leaf (S).

Homozygous *pcap1* and WT plants were inoculated with equal amounts of TuMV-GFP. The level of TuMV-GFP RNA accumulation, as measured by qRT-PCR was not significantly different in both plants at 3 days post inoculation (dpi) (Fig. 7A). The spread of TuMV-GFP in the plant was observed by epifluorescence microscopy (Fig. 7B). In inoculated leaves of *pcap1* plants, at 6 dpi, there were small discrete infection foci (4–6 cells). By 12 dpi, the number of cells in the fluorescent cluster ranged from 15 to 20. However the infection foci were many-fold smaller than in infected WT plants. In WT plants, at both time points, >90% of the infection foci were composed of more than a hundred cells. A dramatic reduction in TuMV-GFP accumulation was also observed in the upper uninoculated second cauline leaf of *pcap1* plants as compared to WT; at 12 dpi, the infection foci in *pcap1* plants contained four to eight cells. The size of the infection foci was reduced greater than 10-fold in *pcap1* plants relative to WT (Fig. 7C). The number of infection foci was also reduced slightly in *pcap1* plants (Fig. 7C).

**Figure 8:**
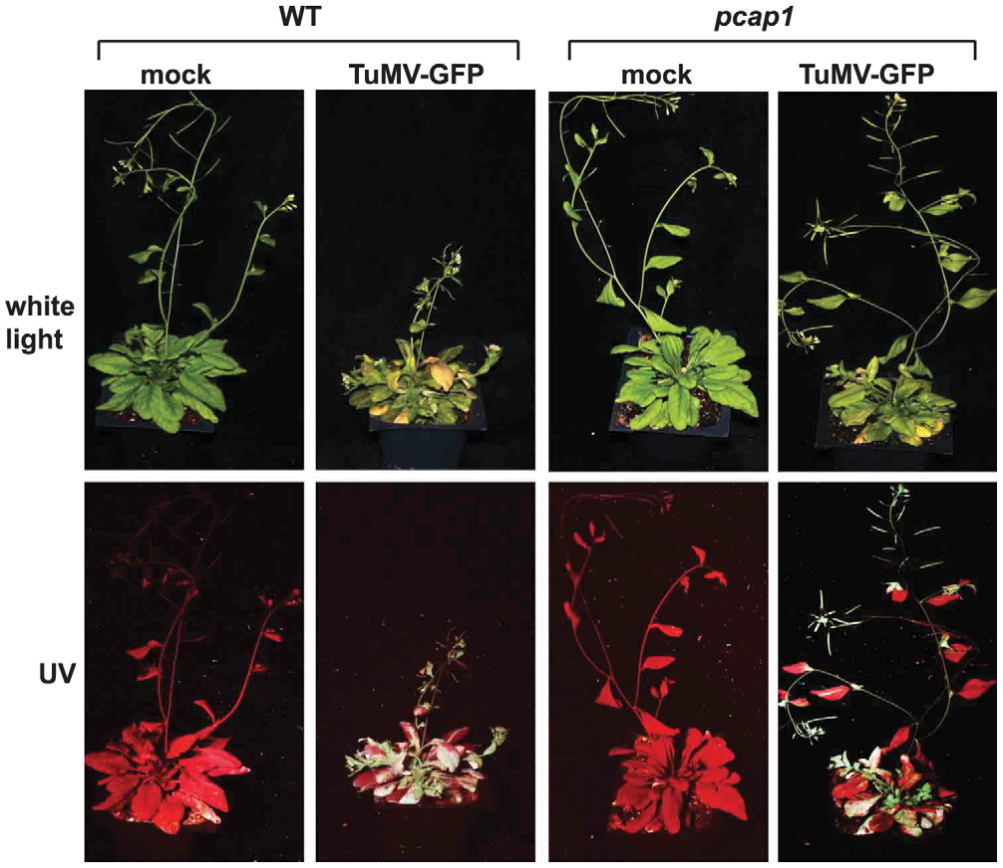
TuMV-GFP infection of wild type and *pcap1 Arabidopsis* plants. Plants were mock inoculated or inoculated with TuMV-GFP and photographed under white light or UV light at 30 dpi. Wild-type (WT), *PCaP1* knockout (*pcap1*).

With regard to disease, in WT plants the symptoms appeared first at 7 dpi and severe symptoms arose systemically in 100% of the inoculated plants. Systemic leaves turned yellow, plants were extremely stunted, and the inflorescence was strongly condensed by 30 dpi (Fig. 8). Most infected plants died. In contrast, in about 70% of the *pcap1* plants, symptoms were delayed 3–4 days in onset and were highly attenuated, showing only mild chlorosis and little to no stunting. By 30 dpi, only very weak GFP fluorescence was observed in young leaves (Fig. 8). Symptoms were less attenuated in about 30% of the inoculated plants but ultimately they showed signs of recovery, unlike WT plants. Uninoculated WT and *pcap1* plants exhibited normal growth (Fig. 8). We conclude that knockout of *PCaP1* greatly reduced the cell-to-cell movement and greatly attenuated but did not completely inhibit TuMV-GFP infection of whole plants.

**Figure 9:**
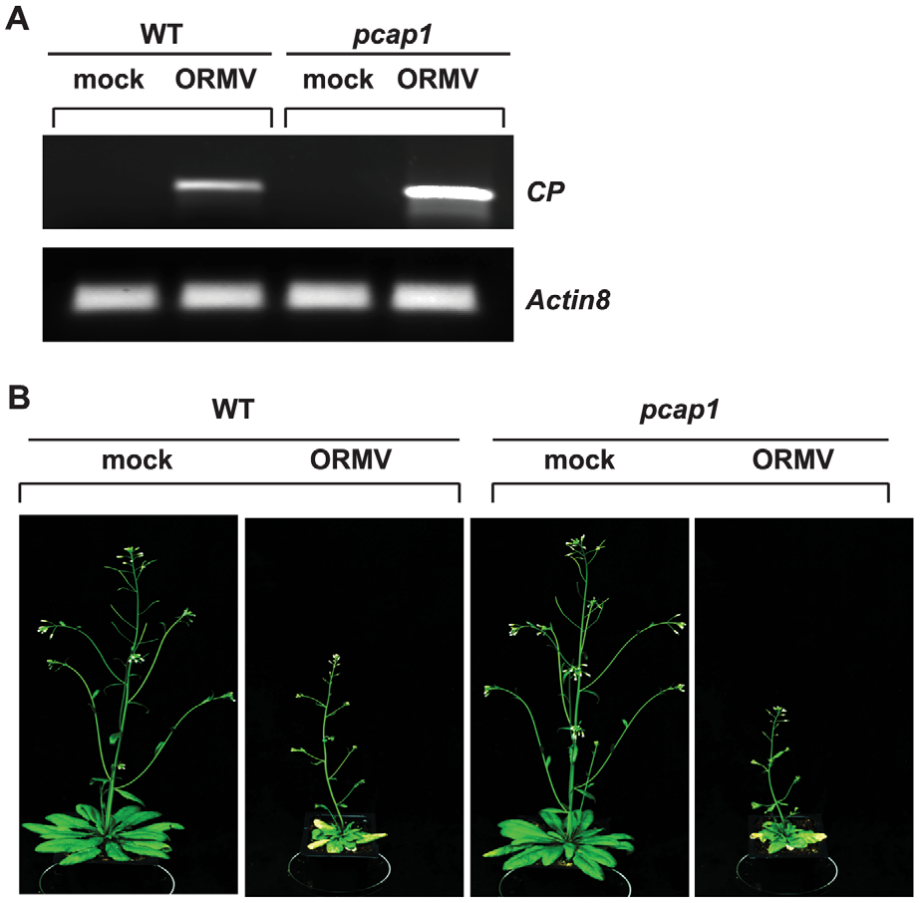
ORMV infection of wild type and *pcap1 Arabidopsis* plants. (A) RT-PCR was used to determine the accumulation of plus-strand ORMV RNAs in upper uninoculated rosette leaves at 6 dpi (top panel). *Actin8* was amplified as control (bottom panel). (B) ORMV infected plants (21 dpi). Wild-type (WT), *PCaP1* knockout (*pcap1*).

To determine whether the requirement for PCaP1 extends beyond potyviruses, we tested the susceptibility of *pcap1* plants to the tobamovirus *Oilseed rape mosaic virus* (ORMV). In upper uninoculated rosette leaves of *pcap1* plants, ORMV plus-strand RNA accumulated similar or slightly more than in WT plants at 6 dpi, as determined by RT-PCR (Fig. 9A). Moreover, *pcap1* plants showed the same stunting and yellowing symptoms as WT plants (Fig. 9B). Thus, PCaP1 is not required for efficient infection by the tobamovirus.

### PCaP1 is not necessary for TuMV-GFP RNA replication

**Figure 10:**
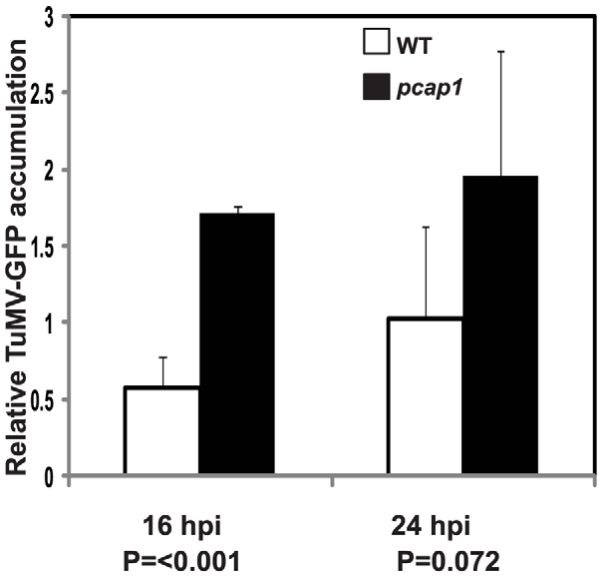
TuMV-GFP RNA accumulation in *Arabidopsis* protoplasts. qRT-PCR was used to quantitate TuMV-GFP RNA accumulation in protoplasts at indicated time points. The values were normalized to the amounts of *Actin8* transcript in the same samples. Data are average of three independent experiments, each consisting of three replicates. Error bars represent standard deviations. Statistical significance between WT and *pcap1* was analyzed by the unpaired Student's *t*-test and calculated P values are indicated.

Finally, we asked if the delayed and reduced accumulation of TuMV-GFP in *pcap1* plants is due to reduced replication of the viral RNA as compared to that in WT plants. Therefore, protoplasts were isolated from leaves of *pcap1* and WT, transfected with TuMV-GFP construct and viral replication was analyzed by qRT-PCR (Fig. 10). Although TuMV-GFP RNA accumulated to a significantly greater level in *pcap1* protoplasts than in WT protoplasts at 16 hours post transfection and by 24 hours there was no significant difference in RNA accumulation. Thus, the delayed virus spread in *pcap1* plants was not likely due to reduced RNA synthesis.

## Discussion

Recognition of a ∼28 kDa polypeptide in TuMV-GFP infected *Arabidopsis* cells by both anti-PIPO and anti-P3 antibodies confirmed our earlier prediction that PIPO is a translational fusion of the N-terminus of P3 and PIPO, and hence the name P3N-PIPO. Mutations in the *pipo* coding region impeded the virus cell-to-cell movement, allowed virus accumulation in cells localized at the inoculation site, and rendered the virus noninfectious or nearly so in whole plants [11], [21], [22]. These observations, combined with our observations that P3N-PIPO-GFP fusion protein mediates its own cell-to-cell movement, strongly support a MP function for P3N-PIPO.

MPs exploit cellular pathways to regulate virus movement by interacting with host factors to change their specific intracellular localization in infected cells, [3], [6], [36], [37]. Y2H screening and two independent plant-based assays, co-IP and BiFC, revealed that P3N-PIPO interacts with the host factor PCaP1. *Arabidopsis* PCaP1 is a hydrophilic cation-binding protein that binds stably to the plasma membrane via N-myristoylation [34]. PCaP1 is constitutively expressed in most organs of the plant, and its expression is increased by elicitors flagellin-oligopeptide, sorbitol or copper [38]. However, PCaP1 protein level was unaffected by TuMV-GFP infection (Fig. 6). This is the first known example of involvement of PCaP1 in virus infection. From several pair-wise interactions in the BiFC assay, it is evident that the PIPO domain of P3N-PIPO interacts directly with PCaP1, and the myristoylation site glycine 2 of PCaP1 is not required for the protein interaction. In the absence of myristoylation, PCaP1 is not expected to be membrane bound. This lack of requirement for membrane binding may explain why the interaction was detected in the Y2H assay that does not detect interactions of integral membrane proteins.

**Figure 11:**
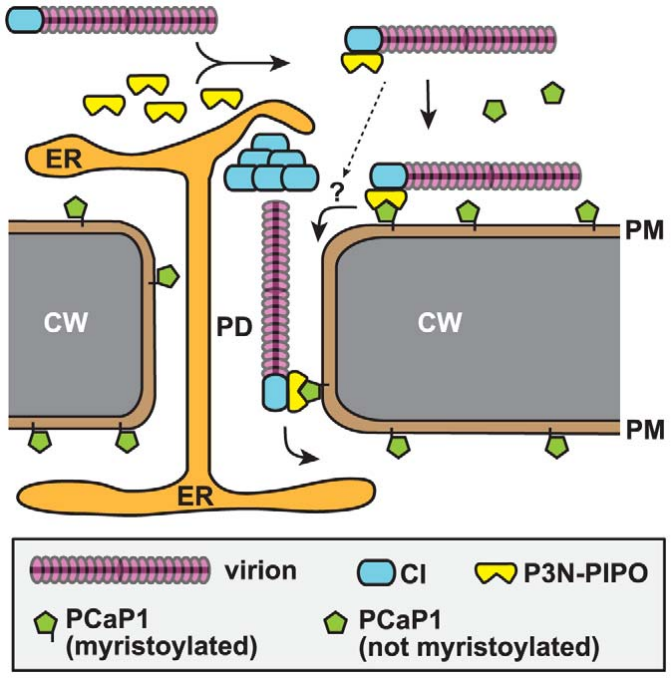
Hypothesis for the role of P3N-PIPO-PCaP1 interaction in potyvirus movement. We speculate that the virion-CI complex binds P3N-PIPO, via the CI protein. P3N-PIPO then transports the complex to the plasma membrane by binding PCaP1, which is anchored to the membrane via its myristoyl moiety. The PCaP1-bound complex accumulates in plasmodesmata possibly by the affinity of CI protein for the plasmodesmata, and/or via unidentified host proteins (question mark). Dashed arrow indicates possible route of inefficient movement of viral movement complex in *PCaP1* knockout plants. ER, endoplasmic reticulum; PD, plasmodesmata; PM, plasma membrane; CW, cell wall.

The interaction of P3N-PIPO with the membrane protein PCaP1 provides a key missing link in the model by which the potyvirus is localized to the plasmodesmata. Carrington et al. [19] provided evidence that an RNP consisting of CI protein bound to CP which is bound to the viral RNA, possibly in the form of a virion, is required for cell-to-cell movement. Wei et al. [23] expanded on this by showing that P3N-PIPO binds CI and moves the RNP complex to plasmodesmata via the secretory pathway. However, neither CP, CI nor P3N-PIPO has a predicted membrane binding or membrane spanning domain, even though membrane binding is expected for movement through the plasmodesmata [6] We hypothesize that the membrane-binding function is provided by PCaP1. By binding P3N-PIPO, PCaP1 may anchor the movement complex to the plasma membrane from which the complex could move to the plasmodesmata (Fig. 11). By virtue of P3N-PIPO's ability to move between cells (Fig. 2), PCaP1-bound P3N-PIPO would move the virion through the plasmodesmata to the neighboring cell (Fig. 11). We also detected P3N-PIPO throughout the cell and in the nucleus in the biolistic bombardment and BiFC assays. Thus, P3N-PIPO may serve other functions and/or the over-expression from a nuclear based expression vector without frameshifting or cleavage from the polyprotein, that are normally required for P3N-PIPO expression, may allow P3N-PIPO to accumulate in areas of the cell not normally occupied in a natural infection. Moreover, this model does not rule out roles for other host proteins in translocating the movement complex to and through the plasmodesmata. Such proteins may facilitate the small amount of local movement observed in the *PCaP1* knockout plants.

The role of PCaP1 in uninfected plants is unknown [38], [39]. It interacts with phosphatidyl inositol phosphates especially PtdIns(3,5)*P2* and PtdIns(3,4,5)*P3*, and calcium-bound calmodulin (Ca^2+^-CAM) complex through an unstructured middle region [34]. Kato et al. [39] propose that binding of Ca^2+^-CAM causes a structural rearrangement in PCaP1 that releases the PtdInsPs, setting off cell signaling. How P3N-PIPO binding to PCaP1 may affect the function and signaling of PCaP1 is unknown. The interaction of P3N-PIPO with PCaP1 may directly affect the calcium levels at the plasmodesmata owing to the Ca^2+^-CAM binding activity of PCaP1. This, in turn, may increase the size exclusion limit (SEL) of the plasmodesmata by reducing callose accumulation. Callose accumulates around the entrance to the plasmodesmata in response to stress and virus infection, and decreases the SEL [40]. Local callose accumulation increases in the presence of Ca^2+^ [41], [42], which increases in a successful defense response to virus infection [43]. Calcium binding proteins have been found to interact with other, unrelated viral MPs. The calcium-modulated synaptotagmin, SYTA, interacts with the MPs of Cabbage leaf curl (CaLCuV) geminivirus and the unrelated TMV [37]. Virus infection is greatly delayed in plants with knockouts of the SYTA gene [37]. The TMV MP also interacts with an ankyrin repeat-containing protein ANK that reduces callose synthesis at the plasmodesmata, increasing the cell-to-cell movement of viral protein by relaxation of callose sphincters [40]. It is possible that the tobamovirus ORMV efficiently infects *pcap1* plants (Fig. 9) because it relies on SYTA and ANK, for similar function(s) provided by PCaP1 to potyviruses. In summary, there is an emerging theme of interaction of viral MPs with calcium-binding proteins, but the role of these interactions in virus movement has yet to be demonstrated.

The fact that viral RNA accumulation was not reduced in protoplasts from *PCaP1* knockout plants indicates that *PCaP1* is not required for the viral RNA synthesis. The apparent 2 to 3-fold increase in viral RNA in the *pcap1* protoplasts relative to that in WT protoplasts leads us to speculate that in *pcap1* protoplasts, the RNA/CP/CI/P3N-PIPO complex would not be localized efficiently to the plasma membrane and thus it would not remove viral RNAs from the vicinity of the replication factories. Instead of being moved to the plasma membrane or plasmodesmata, the newly synthesized RNAs would remain available to act as templates for further rounds of replication. In the presence of PCaP1, the complex would transport to, and accumulate at, the plasma membrane and not be accessible to the replicase for additional rounds of replication.

Finally, *pcap1* mutations or knockouts may provide a new strategy for breeding potyvirus resistance in crop plants. The *pcap1* knockout had no apparent negative effect on phenotype of *Arabidopsis*, although the plants were not tested under different growth conditions or stresses. We detected *PCaP1* orthologs in diverse plant species with identity of up to 67% (Fig. S1). Thus, if *pcap1* knockouts prove not to reduce yield in uninfected plants, knockouts or mutations in this gene could be used for resistance in crop plants. Recessive resistance has been applied widely against economically important Potyviridae in capsicum, lettuce, pea, wheat, barley and other crops and it is generally more durable than dominant resistance genes [44]. In all cases where the resistance gene has been sequenced, it has proven to encode a translation initiation factor [44]. Thus *PCaP1* knockout may represent a potentially new type of potyvirus resistance gene.

## Materials and Methods

All recombinant DNA procedures were carried out by standard methods using *Escherichia coli* strains TOP10, SCS110 (Life Technologies) or Fusion Blue (Clontech) and the clones were verified by sequence analysis.

### Construct for Y2H screening

The TuMV *P3N-PIPO* bait gene was PCR-amplified from pET-P3N-PIPOif, a kind gift from Dr. Betty Chung (University College Cork, Ireland, currently at Cambridge University) which contains an artificial AUG start codon at the beginning of the P3 coding region (normally the cleavage site exists between HC-Pro and P3) and an A insertion in the GGAAAAAA motif to place the *pipo* ORF in-frame with the N-terminal half of the P3 coding region. The PCR primers to amplify the P3N-PIPO sequence from pET-P3N-PIPOif were complementary oligonucleotides P3N-PIPO-2F and P3N-PIPO-2R (Table S1) that introduced *EcoR*I and *Pst*I sites. The *EcoR*I/*Pst*I-digested fragment was cloned into the yeast vector pGBKT7 (Clontech). The plasmid was designated pGBKT7-P3N-PIPO, in which the bait P3N-PIPO gene and GAL4 DNA-binding domain were in-frame.

### Y2H screening of *Arabidopsis* cDNA library

To identify P3N-PIPO interacting host proteins, an *Arabidopsis* (Col-0) cDNA library (Clontech) was screened using the Matchmaker Gold Yeast Two-Hybrid System (Clontech). Briefly, the library strain and Y2HGold strain harboring pGBKT7-P3N-PIPO were mated and plated on double dropout medium containing X- α-Gal (SD/−Leu/−Trp/+X-α-Gal) and incubated at 30°C for 4 days. Cotransformants that were phenotypically positive for α-galactosidase activity were subjected to high stringency screening on quadruple dropout medium SD/-Leu/-Trp/-Ade/-His. Plasmids pGBKT7-T-antigen, pGADT7-laminin C and pGADT7-murine p53 (Clontech) served as controls. Protein interactions were analyzed as described by the manufacturer. From the yeast cells that displayed a positive interaction, prey plasmids were rescued in *E. coli* and sequenced. DNA and protein sequence analyses were performed with the BLAST algorithms (http://blast.ncbi.nlm.nih.gov/Blast.cgi).

### Constructs for cell-to-cell movement and colocalization assays

To construct p35S::P3N-PIPO-GFP, the PCR-amplified P3N-PIPO gene from pET-P3N-PIPOif with primer pair P3N-PIPO-3F/P3N-PIPO-3R (Table S1) was digested with *Sal*I, and cloned in-frame with the GFP ORF between the 35S promoter and *nos* terminator of pJ4GFP-XB (provided by Dr. Diane Bassham, Iowa State University). Plasmid pB7WG2.0.PDCB1-mCherry, which expresses *Arabidopsis* plasmodesmata-specific protein PDCB.1 fused to mCherry [35] was kindly provided by Dr. Andy Maule, John Innes Center, UK.

### Constructs for transient protein expression

P3N-PIPO and PCaP1 were expressed *in planta* with an N-terminal HA or c-myc tag, respectively. The P3N-PIPO gene with HA tag was PCR amplified from pET-P3N-PIPOif with primer pair P3N-PIPO-6F/P3N-PIPO-6R (Table S1), digested with *Bgl*II and *Sal*I, and cloned into the respective sites in the binary vector pMCS11 (a gift from Dr. Diane Bassham) to generate p35S::HA-P3N-PIPO. A c-myc tag was added to PCaP1 gene by PCR amplification from pGADT7-HF3 with primer pair HF3-1F/HF3-1R (Table S1). The primers also added terminal *Bgl*II and *Sal*I sites. The product was digested with *Bgl*II and *Sal*I, and cloned into *Bgl*II-*Sal*I-digested pMCS11 to generate p35S::c-myc-PCaP1.

### Constructs for BiFC assay

Coding sequences of P3N-PIPO, P3N, PIPO, P3, PCaP1 or G2A.PCaP1 were cloned in-frame with the gene encoding N-terminus (YN) or C-terminus (YC) of citrine in the binary vector pSP1823-YN or pSP1794-YC (kindly provided by Dr. S.P. Dinesh-Kumar, University of California, Davis), respectively. To construct p35S::P3N-PIPO-YN, P3N-PIPO, coding sequence was PCR-amplified from pET-P3N-PIPOif with primer pair P3N-PIPO-9F/P3N-PIPO-9R (Table S1). To construct p35S::P3N-YN, p35S::PIPO-YN and p35S::P3-YN, coding region of P3 N-terminus (486 nt), PIPO or full-length P3 was amplified by PCR from p35S::TuMV-GFP with primer pair P3-2F/P3N-1R, PIPO-2F/PIPO-2R or P3-2F/P3-2R (Table S1), respectively. To construct p35S::PCaP1-YC and p35S::G2A.PCaP1-YN, *PCaP1* from pGADT7-HF3 was PCR amplified with primer pair HF3-2F/HF3-5R or HF3-7F/HF3-7R (Table S1). The PCR products were cloned into pENTR-D-TOPO (Life Technologies) and then recombined into the destination vector pSP1823-YN or pSP1794-YC using Gateway LR clonase enzyme mix (Life Technologies).

### Plant growth and genotyping

*A. thaliana* (Col-0) and *N. benthamiana* plants were grown in soil in controlled environment chambers under 16 h light at 22–23°C. Plants of the same age, maintained under identical growth condition were used for each experiment. Genomic DNA was extracted from leaves of *Arabidopsis* T-DNA insertion line SALK_022955 (The *Arabidopsis* Information Resource [45]) using DNeasy Plant Mini kit (Qiagen), and the T-DNA insertion at *PCaP1* was detected using primer pair LB1.3/HF3-RP (Table S1). *PCaP1* in WT plant was verified using primer pair HF3-LP/HF3-RP (Table S1).

### Virus inoculation and DNA delivery

Sap from *Arabidopsis* leaves infected with TuMV infectious clone p35S::TuMV-GFP, or ORMV virion (0.1 mg/ml) (provided by Dr. Steve Whitham, Iowa State University) were used for mechanical inoculations. For biolistic bombardment, detached leaves from four-week old *N. benthamiana* plants were bombarded with either pJ4-GFP-XB or p35S::P3N-PIPO-GFP in a high pressure helium-based PDS-1000 system (Bio-Rad Laboratories) as described previously [33].

### Co-immunoprecipitation

For transient expression of HA-P3N-PIPO or c-myc-PCaP1 *in planta*, p35S::HA-P3N-PIPO or p35S::myc-P3N-PIPO was introduced into *Agrobacterium tumefaciens* GV3101 by electroporation, followed by induction and infiltration into youngest fully-expanded leaves of 3-week old *N. benthamiana* plants [46]. Total proteins from leaves were extracted at 2 days post agroinfiltration in 50 mM Tris-acetate, pH 7.5, 1 mM DTT, 20 µM PMSF, complete protease inhibitor cocktail (Roche) and 1% Triton X-100. An aliquot of the protein extract after centrifugation was used for immunoprecipitation using ProFound HA tag Co-IP kit or c-myc tag Co-IP kit (Thermo Scientific Pierce). Immunocomplexes were recovered in non-reducing sample buffer (Thermo Scientific Pierce) by brief boiling.

### Immunodetection

To detect TuMV-GFP encoded proteins, total proteins from *Arabidopsis* leaves were extracted in 10 mM Tris-HCl, pH 7.4 containing 300 mM NaCl, 5 mM EDTA, 1 mM PMSF and 1 mM DTT. To detect PCaP1, total proteins were extracted as described in [38] and solubilized in 1% Triton X-100. The proteins were separated in Novex 4–12% Tris-glycine gel (Life Technologies) unless otherwise stated, electroblotted on to a polyvinylidene diflouride (PVDF) membrane (Bio-Rad) using Western blotting reagents (AMERCO, USA) and probed with respective antibodies. Rabbit anti-PIPO serum for an N-terminal peptide representing amino acids 2–15 of PIPO [11] and anti-P3 serum generated against an oligopeptide representing amino acids 3–16 of P3 (GenScript, USA) were used at 1/5000 dilution. Rabbit polyclonal anti-GFP antibodies (Life Technologies) were used at dilution of 1/1000. Rabbit anti-PCaP1 antibodies developed against oligopeptide representing amino acids 152–166 of PCaP1 [38] (Cosmo Bio, Japan) were used at 1∶2000 dilution. Rabbit anti-HA polyclonal and mouse anti-c-myc monoclonal antibodies (Clontech) were used as suggested by the manufacturer. The blots were developed with anti-rabbit antibody (Thermo Scientific) or anti-mouse antibody (AMRESCO) conjugated to horse-radish peroxidase and detected using the ECL-Plus Western blotting reagents (GE Health Care).

### BiFC and colocalization assays

Proteins fused to the N- or C- terminal half of citrine were expressed in young leaves of 3–4 week old *N. benthamiana* plants by infiltration of *A. tumefaciens* GV3101 harboring the appropriate plasmid, as described by Hayward et al. [46]. In colocalization assay, PDCB1-mCherry was co-expressed. To avoid overexpression of recombinant proteins in both assays, *Agrobacterium* was used at A_600_ 0.05. At about 38 h post agroinfiltration, leaves were analyzed for BiFC or colocalization by confocal microscopy. Construct pSP862 (provided by Dr. S.P. Dinesh-Kumar) that has a GUS gene in fusion with YC served as negative control.

### RT-PCR and qRT-PCR

Total RNA was extracted from healthy or virus infected *Arabidopsis* leaves or protoplasts using the RNasy plant minikit (Qiagen) and treated with RNase-free DNase I (Ambion). RT-PCR was performed with SuperScript III One Step RT-PCR system with Platinum Taq (Life Technologies) and ORMVCP-1F/ORMVCP-1R primer pair (Table S1). For qRT-PCR analysis, 30 ng of total RNA and gene-specific primers at a concentration of 10 pmol were used. For TuMV quantification, HC-Pro gene-specific primer pair TuHCqRT-F/TuHCqRT-R (Table S1) was used. TuMV accumulation in each sample was normalized to the quantity of *Actin8* that was amplified with primer pair Actin8qRT-F/Actin8qRT-R (Table S1). qRT-PCR was performed using qScript One-Step SYBR Green qRT-PCR kit (Quanta Biosciences, USA) according to the manufacturer's recommendations in the iCycler IQ system (Bio-Rad Laboratories). PCR cycles were as follows: 1 cycle of 10 min at 50°C and 5 min at 95°C, followed by 45 cycles each of 10 sec at 94°C and 30 s at 61°C. A dissociation curve was produced at the end of the cycling phase to ensure that a single PCR product was produced with no primer dimers. Absolutequantification was performed using the standard curve method. Data were statistically evaluated by the unpaired Student's *t*-test.

### Protoplast isolation and TuMV replication assay

Mesophyll protoplasts were prepared from 4 week-old WT and *pcap1 Arabidopsis* leaves by the procedure of Yoo et al [47]. The leaves were sterilized in 30% ethanol for 1 min, followed by rinses in distilled water and incubated in 1–1.5% cellulase Onozuka R-10 (Yakult Pharamaceuticals, Japan) and 0.2–0.4% macerozyme R-10 (Yakult Honsha, Japan) in 0.4 M D-sorbitol, 20 mM KCl and 20 mM MES, pH 5.7 for 2 h at room temperature. Protoplasts were washed in W5 buffer (154 mM NaCl, 125 mM CaCl_2_, 5 mM KCl and 2 mM MES, pH 5.7) and resuspended in MMG buffer (0.4 M mannitol, 15 mM MgCl_2_ and 4 mM MES pH 5.7) at a concentration of 10^5^ cells/ml. Protoplasts were transfected with 10 µg of p35::TuMV-GFP in 40% PEG 4000 in 0.8 M mannitol and 1 M CaCl_2_ at room temperature for 30 min. Transformed protoplasts were then washed, resuspended in W5 buffer and incubated for virus to replicate.

### Microscopy

Leaves expressing recombinant proteins were imaged using a Leica SP5 X inverted confocal microscope with an Argon laser. GFP was excited at 484 nm and the emitted light was captured at 507 nm. Citrine was excited at 514 nm and the emitted light was captured at 527 nm. mCherry was excited at 587 nm and the emission was captured at 610 nm. Images were captured digitally and processed using Leica Application Suite 2.3.0. All fluorescent images are projections of optical sections.

TuMV infection foci were assessed with Zeiss Axioscope 20 fluorescence microscope and images were acquired either with a Nikon Eclipse DXM1200 digital camera (mounted on the Nikon Eclipse E800) and Nikon ACT-1 software. Size of the infection was measured by Analysis Pro software (Olympus Imaging System, Olympus Corpn., USA). Images were processed using Adobe Photoshop CS5 software (Adobe Systems Inc.).

### GUS staining

GUS expression in *N. benthamiana* leaves was detected by histochemical staining [48].

### Sequence alignment and accession numbers

Sequences of PCaP1 orthologs were aligned using ClustalW (http://www.ebi.ac.uk/Tools/msa/clustalw2/) and processed in Jalview version 2 [49]. The GenBank accession number for the *Arabidopsis* PCaP1 sequence reported in this paper is NP_001031677. The accession numbers for the PCaP1 orthologs are: *Medicago truncatula*; ACJ84038, *Vitis vinifera*; XP_002263090, *Nicotiana tabacum*; CAB91552, *Glycine max*; XP_003546380, *Cicer arietinum*; CAB61742, *Ricinus communis*; XP_002532713, *Oryza sativa*; NP_001046572, *Zea mays*; NP_001150000, *Sorghum bicolor*; XP_002453713, *Picea sitchensis* ABK21073.

## Supporting Information

Figure S1 **Amino acid sequence alignment of PCaP1 proteins from** ***Arabidopsis*** **and other plant species.** The PCaP1 from *A. thaliana* (accession number NP_001031677) was aligned, as described in Methods, with its orthologs from *Medicago truncatula* (*Medicago*, accession number ACJ84038), *Vitis vinifera* (*Vitis*, accession number XP_002263090), *Nicotiana tabacum* (*Nicotiana*, accession number CAB91552), *Glycine max* (*Glycine*, accession number XP_003546380), *Cicer arietinum* (*Cicer*, accession number CAB61742), *Ricinus communis* (*Ricinus*, accession number XP_002532713), *Oryza sativa* (*Oryza*, accession number NP_001046572), *Zea mays* (*Zea*, accession number NP_001150000), *Sorghum bicolor* (*Sorghum*, accession number XP_002453713), *Picea sitchensis* (*Picea*, accession number ABK21073). Notice that the glycine at position 2, which is the N-myristoylation site in *Arabidopsis* PCaP1, is conserved only in the dicot species (indicated by vertical green line on the left) but neither in the monocots (red line) nor the gymnosperm (black line). Identical amino acid residues between PCaP1 orthologs are highlighted.
(TIF)

Table S1 **Primer sequences used in this study.** HF3 (PCaP1), TuHC (TuMV HC-Pro), F (forward), R (Reverse), LP, RP and LB (primers used for genotyping).
(DOC)

## References

[1] Lucas WJ, Ham BK, Kim JY (2009). Plasmodesmata - bridging the gap between neighboring plant cells.. Trends Cell Biol.

[2] Maule AJ, Benitez-Alfonso Y, Faulkner C (2011). Plasmodesmata - membrane tunnels with attitude.. Curr Opin Plant Biol.

[3] Boevink P, Oparka KJ (2005). Virus-host interactions during movement processes.. Plant Physiol.

[4] Ueki S, Citovsky V (2011). To Gate, or Not to Gate: Regulatory Mechanisms for Intercellular Protein Transport and Virus Movement in Plants.. Mol Plant.

[5] Verchot-Lubicz J, Torrance L, Solovyev AG, Morozov SY, Jackson AO (2010). Varied movement strategies employed by triple gene block-encoding viruses.. Mol Plant Microbe Interact.

[6] Niehl A, Heinlein M (2011). Cellular pathways for viral transport through plasmodesmata.. Protoplasma.

[7] Harries P, Ding B (2011). Cellular factors in plant virus movement: at the leading edge of macromolecular trafficking in plants.. Virology.

[8] Laporte C, Vetter G, Loudes AM, Robinson DG, Hillmer S (2003). Involvement of the secretory pathway and the cytoskeleton in intracellular targeting and tubule assembly of Grapevine fanleaf virus movement protein in tobacco BY-2 cells.. Plant Cell.

[9] Riechmann JL, Lain S, Garcia JA (1992). Highlights and prospects of potyvirus molecular biology.. J Gen Virol.

[10] Urcuqui-Inchima S, Haenni AL, Bernardi F (2001). Potyvirus proteins: a wealth of functions.. Virus Res.

[11] Chung BY-W, Miller WA, Atkins JF, Firth AE (2008). An overlapping essential gene in the Potyviridae.. Proc Natl Acad Sci U S A.

[12] Shen W, Yan P, Gao L, Pan X, Wu J (2010). Helper component-proteinase (HC-Pro) protein of Papaya ringspot virus interacts with papaya calreticulin.. Mol Plant Pathol.

[13] Dolja VV, Haldeman R, Robertson NL, Dougherty WG, Carrington JC (1994). Distinct functions of capsid protein in assembly and movement of tobacco etch potyvirus in plants.. EMBO J.

[14] Dolja VV, Haldeman-Cahill R, Montgomery AE, Vandenbosch KA, Carrington JC (1995). Capsid protein determinants involved in cell-to-cell and long distance movement of tobacco etch potyvirus.. Virology.

[15] Rojas MR, Zerbini FM, Allison RF, Gilbertson RL, Lucas WJ (1997). Capsid protein and helper component-proteinase function as potyvirus cell-to-cell movement proteins.. Virology.

[16] Hofius D, Maier AT, Dietrich C, Jungkunz I, Bornke F (2007). Capsid protein-mediated recruitment of host DnaJ-like proteins is required for Potato virus Y infection in tobacco plants.. J Virol.

[17] Rodriguez-Cerezo E, Findlay K, Shaw JG, Lomonossoff GP, Qiu SG (1997). The coat and cylindrical inclusion proteins of a potyvirus are associated with connections between plant cells.. Virology.

[18] Dunoyer P, Thomas C, Harrison S, Revers F, Maule A (2004). A cysteine-rich plant protein potentiates Potyvirus movement through an interaction with the virus genome-linked protein VPg.. J Virol.

[19] Carrington JC, Jensen PE, Schaad MC (1998). Genetic evidence for an essential role for potyvirus CI protein in cell-to-cell movement.. Plant J.

[20] Roberts IM, Wang D, Findlay K, Maule AJ (1998). Ultrastructural and temporal observations of the potyvirus cylindrical inclusions (Cls) show that the Cl protein acts transiently in aiding virus movement.. Virology.

[21] Choi IR, Horken KM, Stenger DC, French R (2005). An internal RNA element in the P3 cistron of Wheat streak mosaic virus revealed by synonymous mutations that affect both movement and replication.. J Gen Virol.

[22] Wen RH, Hajimorad MR (2010). Mutational analysis of the putative pipo of soybean mosaic virus suggests disruption of PIPO protein impedes movement.. Virology.

[23] Wei T, Zhang C, Hong J, Xiong R, Kasschau KD (2010). Formation of complexes at plasmodesmata for potyvirus intercellular movement is mediated by the viral protein P3N-PIPO.. PLoS Pathog.

[24] Gabrenaite-Verkhovskaya R, Andreev IA, Kalinina NO, Torrance L, Taliansky ME (2008). Cylindrical inclusion protein of potato virus A is associated with a subpopulation of particles isolated from infected plants.. J Gen Virol.

[25] Anandalakshmi R, Marathe R, Ge X, Herr JM, Mau C (2000). A calmodulin-related protein that suppresses posttranscriptional gene silencing in plants.. Science.

[26] Guo D, Spetz C, Saarma M, Valkonen JP (2003). Two potato proteins, including a novel RING finger protein (HIP1), interact with the potyviral multifunctional protein HCpro.. Mol Plant Microbe Interact.

[27] Ballut L, Drucker M, Pugniere M, Cambon F, Blanc S (2005). HcPro, a multifunctional protein encoded by a plant RNA virus, targets the 20S proteasome and affects its enzymic activities.. J Gen Virol.

[28] Dielen AS, Sassaki FT, Walter J, Michon T, Menard G (2011). The 20S proteasome alpha5 subunit of Arabidopsis thaliana carries an RNase activity and interacts in planta with the lettuce mosaic potyvirus HcPro protein.. Mol Plant Pathol.

[29] Jin Y, Ma D, Dong J, Li D, Deng C (2007). The HC-pro protein of potato virus Y interacts with NtMinD of tobacco.. Mol Plant Microbe Interact.

[30] Cheng YQ, Liu ZM, Xu J, Zhou T, Wang M (2008). HC-Pro protein of sugar cane mosaic virus interacts specifically with maize ferredoxin-5 in vitro and in planta.. J Gen Virol.

[31] Gao Z, Johansen E, Eyers S, Thomas CL, Noel Ellis TH (2004). The potyvirus recessive resistance gene, sbm1, identifies a novel role for translation initiation factor eIF4E in cell-to-cell trafficking.. Plant J.

[32] Lucas WJ (2006). Plant viral movement proteins: agents for cell-to-cell trafficking of viral genomes.. Virology.

[33] Vijaya Palani P, Kasiviswanathan V, Chen JC, Chen W, Hsu YH (2006). The arginine-rich motif of Bamboo mosaic virus satellite RNA-encoded P20 mediates self-interaction, intracellular targeting, and cell-to-cell movement.. Molecular plant-microbe interactions : Mol Plant Microbe Interact.

[34] Nagasaki N, Tomioka R, Maeshima M (2008). A hydrophilic cation-binding protein of Arabidopsis thaliana, AtPCaP1, is localized to plasma membrane via N-myristoylation and interacts with calmodulin and the phosphatidylinositol phosphates PtdIns(3,4,5)P(3) and PtdIns(3,5)P(2).. FEBS J.

[35] Simpson C, Thomas C, Findlay K, Bayer E, Maule AJ (2009). An Arabidopsis GPI-anchor plasmodesmal neck protein with callose binding activity and potential to regulate cell-to-cell trafficking.. Plant Cell.

[36] Whitham SA, Wang Y (2004). Roles for host factors in plant viral pathogenicity.. Curr Opin Plant Biol.

[37] Lewis JD, Lazarowitz SG (2010). Arabidopsis synaptotagmin SYTA regulates endocytosis and virus movement protein cell-to-cell transport.. Proc Natl Acad Sci USA.

[38] Ide Y, Nagasaki N, Tomioka R, Suito M, Kamiya T (2007). Molecular properties of a novel, hydrophilic cation-binding protein associated with the plasma membrane.. J Exp Bot.

[39] Kato M, Nagasaki-Takeuchi N, Ide Y, Tomioka R, Maeshima M (2010). PCaPs, possible regulators of PtdInsP signals on plasma membrane.. Plant Signal Behav.

[40] Ueki S, Spektor R, Natale DM, Citovsky V (2010). ANK, a host cytoplasmic receptor for the Tobacco mosaic virus cell-to-cell movement protein, facilitates intercellular transport through plasmodesmata.. PLoS Pathog.

[41] Furch AC, Zimmermann MR, Will T, Hafke JB, van Bel AJ (2010). Remote-controlled stop of phloem mass flow by biphasic occlusion in Cucurbita maxima.. J Exp Bot.

[42] Dangl JL, Dietrich RA, Richberg MH (1996). Death Don't Have No Mercy: Cell Death Programs in Plant-Microbe Interactions.. Plant Cell.

[43] Otulak K, Garbaczewska G (2011). Cellular localisation of calcium ions during potato hypersensitive response to Potato virus Y.. Micron.

[44] Truniger V, Aranda MA (2009). Recessive resistance to plant viruses.. Adv Virus Res.

[45] Alonso JM, Stepanova AN, Leisse TJ, Kim CJ, Chen H (2003). Genome-wide insertional mutagenesis of Arabidopsis thaliana.. Science.

[46] Hayward A, Padmanabhan M, Dinesh-Kumar SP (2011). Virus-induced gene silencing in nicotiana benthamiana and other plant species.. Methods Mol Biol.

[47] Yoo SD, Cho YH, Sheen J (2007). Arabidopsis mesophyll protoplasts: a versatile cell system for transient gene expression analysis.. Nat Protoc.

[48] Jefferson RA (1987). Assaying chimeric genes in plants: The GUS gene fusion system.. Plant Mol Biol.

[49] Waterhouse AM, Procter JB, Martin DMA, Clamp M, Barton GJ (2009). Jalview Version 2-a multiple sequence alignment editor and analysis workbench.. Bioinformatics.

