# Supplementary figures and images for: Interaction of the Trans-Frame Potyvirus Protein P3N-PIPO with Host Protein PCaP1 Facilitates Potyvirus Movement
Source: PLoS Pathog. 2012 Apr 12;8(4):e1002639. doi: 10.1371/journal.ppat.1002639 (PMC3325209; doi:10.1371/journal.ppat.1002639)

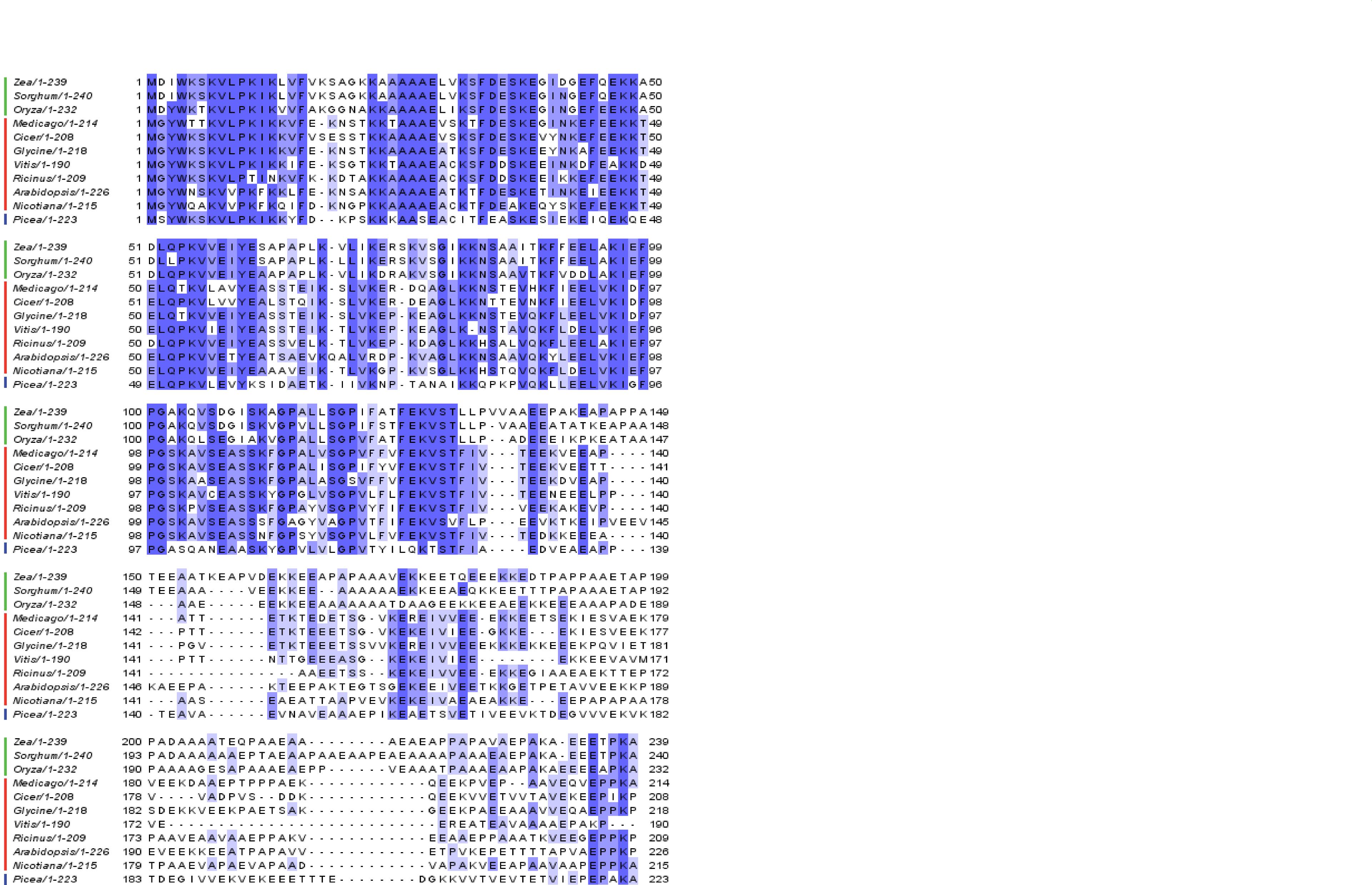

Supplement: Figure S1 — Amino acid sequence alignment of PCaP1 proteins from Arabidopsis and other plant species. The PCaP1 from A. thaliana (accession number NP_001031677) was aligned, as described in Methods, with its orthologs from Medicago truncatula (Medicago, accession number ACJ84038), Vitis vinifera (Vitis, accession number XP_002263090), Nicotiana tabacum (Nicotiana, accession number CAB91552), Glycine max (Glycine, accession number XP_003546380), Cicer arietinum (Cicer, accession number CAB61742), Ricinus communis (Ricinus, accession number XP_002532713), Oryza sativa (Oryza, accession number NP_001046572), Zea mays (Zea, accession number NP_001150000), Sorghum bicolor (Sorghum, accession number XP_002453713), Picea sitchensis (Picea, accession number ABK21073). Notice that the glycine at position 2, which is the N-myristoylation site in Arabidopsis PCaP1, is conserved only in the dicot species (indicated by vertical green line on the left) but neither in the monocots (red line) nor the gymnosperm (black line). Identical amino acid residues between PCaP1 orthologs are highlighted. (TIF) [file ppat.1002639.s001.tif]
