# Supplementary material for: Interaction of the Trans-Frame Potyvirus Protein P3N-PIPO with Host Protein PCaP1 Facilitates Potyvirus Movement
Source: PLoS Pathog. 2012 Apr 12;8(4):e1002639. doi: 10.1371/journal.ppat.1002639 (PMC3325209; doi:10.1371/journal.ppat.1002639)
Supplement: Table S1 — Primer sequences used in this study. HF3 (PCaP1), TuHC (TuMV HC-Pro), F (forward), R (Reverse), LP, RP and LB (primers used for genotyping). (DOC) [file ppat.1002639.s002.doc]

| **Gene** | **Primer** |
| --- | --- |
| P3N-PIPO-2F | GCGGATCCGAATTCGGAACAGAATGGGAG |
| P3N-PIPO-2R | GACGGAGCTCGAACTGCAGTTACTCCGTTCGTAAGATG |
| P3N-PIPO-3F | GGTCGCGGAGTCGACATGGGAACAGAATGGGAG |
| P3N-PIPO-3R | TGTCGACGGAGCTCGTCGACCTCCGTTCGTAAGATG |
| P3N-PIPO-6F | GTGCCGCGCGGCAGCCATATGGCTAGCATGAGATCTATGTA CCCATACGATGTTCCAGATTACGCTGGAACAGAATGGGAG |
| P3N-PIPO-6R | TTGTCGACGGAGGTCGACTCACTCCGTTCGTAAGATGAC |
| P3N-PIPO-9F | CACCATGGGAACAGAATGGGA |
| P3N-PIPO-9R | CTCCGTTCGTAAGATGACATG |
| PIPO-2F | CACCATGGGAAAAAAGTTATCT |
| PIPO-2R | CTCCGTTCGTAAGATGACATG |
| P3N-1R | CAAAATGGAGATGCTATGATC |
| P3-2F | CACCATGGGAACAGAATGGGAG |
| P3-2R | TTGCTTACTCCGTTCGTAAGA |
| HF3-1F | GTTTCAAAGATCTATGGCATCAATGCAGAAGCTGATGGACCTGATCT  CAGAGGAGGGTTACTGGAAT |
| HF3-1R | TAATCATACCATCTTGAAGTCGACTCAAGGCTTTGGTGG |
| HF3-2F | CACCATGGGTTACTGGAATTCC |
| HF3-5R | AGGCTTTGGTGGTTCAGC |
| HF3-7F | CACCATGGCTTACTGGAATTCC |
| HF3-7R | AGGCTTTGGTGGTTCAGCCACTGG |
| HF3-LP | AAATCAAGGCTTTGGTGGTTC |
| HF3-RP | CTAAAATGGGCACGTATGGTG |
| LB1.3 | ATTTTGCCGATTTCGGAAC |
| ORMVCP-1F | CAGAGCCCACAGCGATGC |
| ORMVCP-1R | CCTCGAACTCTGCCCTGT |
| TuHCqRT-F | GGAGCCAACTTCTGGAAAG |
| TuHCqRT-R | GGTTATCTTTCCGCATGGGAACA |
| Actin8qRT-F | ccatgacgggatcacatttc |
| Actin8qRT-R | caaacgctgtaaccggaaag |
